# Complete Systematic Error Model of SSR for Sensor Registration in ATC Surveillance Networks

**DOI:** 10.3390/s17102171

**Published:** 2017-09-21

**Authors:** Ángel J. Jarama, Jaime López-Araquistain, Gonzalo de Miguel, Juan A. Besada

**Affiliations:** Signals, Systems and Radiocommunications Department, Universidad Politécnica de Madrid, 28040 Madrid, Spain; jaime.lopez@grpss.ssr.upm.es (J.L.-A.); gonzalo@grpss.ssr.upm.es (G.d.M.); besada@grpss.ssr.upm.es (J.A.B.)

**Keywords:** bias estimation, radar modeling, measurement error, air traffic control, sensor fusion

## Abstract

In this paper, a complete and rigorous mathematical model for secondary surveillance radar systematic errors (biases) is developed. The model takes into account the physical effects systematically affecting the measurement processes. The azimuth biases are calculated from the physical error of the antenna calibration and the errors of the angle determination dispositive. Distance bias is calculated from the delay of the signal produced by the refractivity index of the atmosphere, and from clock errors, while the altitude bias is calculated taking into account the atmosphere conditions (pressure and temperature). It will be shown, using simulated and real data, that adapting a classical bias estimation process to use the complete parametrized model results in improved accuracy in the bias estimation.

## 1. Introduction

Nowadays radars are the basic element in Air Traffic Control (ATC) networks. Modern sensors as Wide Area Multilateration (WAM) [1] and Automatic Dependent Surveillance-Broadcast (ADS-B) [2] have higher performance than radars with lower cost, so in the future it’s expected they will partially take the place of radars. In any case, radars will still be used as a backup network for many years. The type of radar most used in ATC sensor network is the Secondary Surveillance Radar (SSR), being Mode S radar an enhanced version of this system [3]. SSR is a rotating 2D radar sending an interrogation though an antenna with high directivity in azimuth and low directivity in elevation (2D antenna). Afterwards the aircraft transponder replies with an answer codifying barometric altitude (Mode C) and identification (Mode S and/or Mode A) data. SSR radars estimate the position of the aircraft in polar coordinates, using the distance, the azimuth (both measured by the radar) and the altitude (measured by an airborne barometric altimeter).

To enable surveillance of wide areas radars with overlapped coverages are deployed. Therefore, aircraft measures (plots) from different sensors are sent to a control center where the data from all sensors is fused in order to get a unique trajectory estimation (track) for each aircraft. All the measures are transformed from radar polar local coordinates to a common coordinated system, usually Cartesian coordinates projected over the stereographic plane [4]. To make this data fusion process stable and accurate, the systematic errors affecting the measures from each radar must be corrected before the change of coordinates. If this correction isn’t applied the measures of the same aircraft from different sensors are misaligned and the track could be either very unstable (apparent zig-zag maneuvers induced by measurements misalignment), or in extreme cases it could even be split into several tracks (i.e., one per sensor).

Radar data fusion systems usually have an algorithm that estimates the misalignment error using the measures from the current aircraft in the airspace (opportunity traffic). These algorithms are usually based in the least squares (LS) method (or similar approaches, such as WLS, or MSE estimators [5]) and have the need of a parametrized mathematical model that links the systematic errors in azimuth, distance and altitude with the sensor physical non-idealities.

In the abundant literature about biases estimation methods in ATC networks such as [6,7,8], the mathematical bias models used are simple (modeling just a range and azimuth offset and a range bias term proportional to range) because the objective of the papers was showing the estimation methods. Recently in [9] a novel registration algorithm has been presented where the track state and sensor biases are estimated simultaneously modeling the sensors with range and azimuth offset. Other related examples can be found in [10,11]. In real applications, these models are too simple, resulting in reduced performance when used with real data. At the same time, the development of mathematical models for biases is a classic topic for radio electric signals. For instance, in [12] a model is developed for measurement errors in azimuth for antennas in Deep Space Networks. Meanwhile, [13,14,15,16,17] it’s developed an azimuth bias model for 3D antennas. Due to the dispersion of the literature about the systematic error modelling, it is apparent quite often both in the literature and in real systems there is much effort on improving estimation processes but little effort is given to rigorous modelling of the error sources.

The objective of this paper is to derive a complete systematic error model for ATC radars enabling an important improvement of the bias estimation processes. In Section 2 we will introduce a measurement model for azimuth, distance and altitude measurements. Then, in Section 3, Section 4 and Section 5, we will derive the relation between the measurement error and the measurement biases terms. Section 6 describes a simple bias estimation process (based on opportunity traffic) to be used to show the improvement in estimation due to improved error modelling. Then, Section 7 includes simulation and real data results showing the aforementioned improvement. In the results it is shown the improvement of the alignment that can be obtained using a complete model in comparison with a simple model. Finally, Section 8 concludes the paper and gives some clues about future research.

## 2. Measurement Model

Radars used in ATC typically use a two dimensional rotating antenna and have only the capability to measure aircraft range and azimuth. The azimuth of the target is the angle of the antenna boresight while the target altitude is measured by the aircraft navigation system and can be communicated to radar through a data link. Primary Surveillance Radars (PSR) only can determine the range and the azimuth of the target due to the lack of data link capability. Nowadays in ATC systems primary radars are used as backup system to secondary radar network. SSR is based in the same concept that PSR but in this case, the SSR has data link capability. SSR sends an interrogation message and the aircraft responds with its Mode A/Mode S (identifying code) or mode C (barometric altitude).

The azimuth and the range of the target are determined respectively with antenna boresight (usually corrected using monopulse [3]) and the time lapse between the radar interrogation and its response. Range and azimuth are typically referenced to a local Cartesian coordinated system: *y*-axis pointing to north, *x*-axis pointing to East and *z*-axis pointing to up (ENU system). Azimuth reference has its origin in the *y*-axis and it grows in clockwise. Barometric altitude is measured by an airborne barometric altimeter with reference at mean sea level. The relation between radar coordinates ($\rho_{m},\;\theta_{m},\;h_{m}$) and local Cartesian coordinates ($x_{m},y_{m}$) is:

(1) $$x_{m}=\rho_{m}\sin\left(\theta_{m}\right)\cos\left(\varphi_{m}\right)\;y_{m}=\rho_{m}\cos\left(\theta_{m}\right)\cos\left(\varphi_{m}\right)$$

where $\rho_{m},\;\theta_{m},\;h_{m}$ are the range, azimuth and altitude of the measurement and $\varphi_{m}$ is the target elevation with respect to *i*-th sensor horizontal plane, which can be calculated as:

(2) $$\varphi_{m}=arcsin\left(\frac{2R\left(h_{m}-h_{i}\right)+h_{m}^{2}-h_{i}^{2}-\rho_{m}^{2}}{2\rho_{m}\left(R+h_{i}\right)}\right)$$

being $h_{i}$ the geometric altitude of the *i*-th sensor with reference at mean sea level. *R* is the local Earth radius at the radar position, calculated as described in [18]. Local Cartesian coordinates will be used as middle step in the conversion between radar measures and common tracking coordinates for all sensors.

The radar measurement model can be summarized as:

(3) $$\rho_{m}=\rho +n_{\rho }+\Delta \rho \;\theta_{m}=\theta +n_{\theta }+\Delta \theta \;h_{m}=h+n_{h}+\Delta h$$

where $n_{\rho }$, $n_{\theta }$ and $n_{h}$ are the noise measurement errors for range, azimuth and altitude respectively, Δρ, Δθ and Δh are systematic error terms due to bad system calibration, and ρ, θ and h are the ideal range, azimuth and altitude of the target. Noise terms are modelled typically as uncorrelated additive white noises (usually considered Gaussian distributed), while systematic error terms can be considered constants or slow time variant (bias terms). Bias terms of range, azimuth and altitude depend on target position and will be deduced in Section 3, Section 4 and Section 5 for SSR sensors. They are composed by several environmental and equipment perturbations that are common to all the measurements of each sensor. The parameters of the mathematical model may change with time, due to variable weather conditions and hardware aging.

In surveillance data fusion systems the measures obtained from different kind of sensor are fused in order to improve the information update ratio and the accuracy of the aircraft trajectory estimation. At the moment of the data fusion all measurement must be expressed in a common coordinated reference system. There are several coordinated systems that can be used in ATC. For small scenarios, flat Earth model can be assumed, therefore Cartesian coordinates are precise enough to track the trajectories with several sensors. When the scenario is larger and sensors are widely separated, the Earth curvature must be considered and a different coordinate system must be used. A typical solution is to use stereographic projection to project all measures expressed in local Cartesian reference systems to the same plane [4]. Afterwards, the multisensor tracking process will be performed in the stereographic reference system for horizontal track. Aircraft barometric altitude is usually tracked separately in ATC applications.

## 3. Azimuth Bias

Azimuth bias can be modelled as the superposition of several terms that can be separated in three different groups. The first one is the erroneous orientation of the antenna boresight due to misalignments between the antenna and the rotation axis. The second one is the non-orthogonality between the rotation axis and the Earth surface. And the last one is the calibration error of the dispositive of angle determination (azimuth encoder). Some of these terms will have the same effect in the measures and they cannot be distinguished in the estimation process using measures from radar. For example, the azimuth offset of the antenna, the azimuth offset of the rotation axis and the azimuth offset of the encoder will have equivalent effects and only one of them will be considered. In this case, the model adds all the effects in a single parameter.

In other cases, the superposition of these terms will depend on aircraft position. Antenna skew and rotation axis skew will be distinguishable if the aircraft are distributed in azimuth. For these two bias terms both the elevation and azimuth diversity of the aircraft are needed in order to estimate them. Generally, the aircraft will be distributed in the airspace (position and altitude) but sometimes there will be cases where all the aircraft will be concentrated in a small rank of azimuth, altitude or range. In these cases, the bias estimation algorithms will not have the capability to separate the different azimuth and bias components (there would be an observability problem [15]).

### 3.1. Antenna Deviation

The first factor that affects the azimuth bias is the deviation between the vertical axis of the antenna reference and the reference of the rotation axis. This deviation will be defined with three parameters describing the rotation in the three axes of the Cartesian coordinate system. In order to determine the antenna deviation, the coordinated system is defined as: *y*-axis, parallel to the ideal antenna boresight; *z*-axis, orthogonal to the *y*-axis, resulting that both axes compose the ideal plane of the 2D antenna pattern (*z*-axis is the ideal antenna rotation axis); the *x*-axis, orthogonal to the *y*-axis and *z*-axis positive to the right.

**Figure 1:**
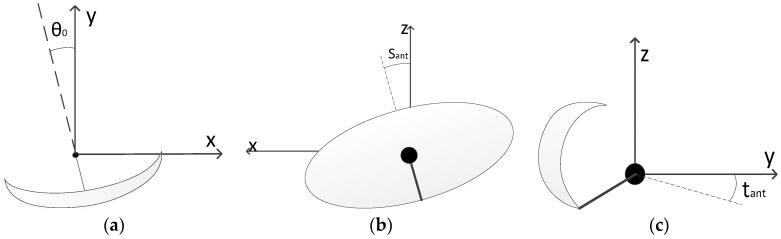
Representation of: (**a**) antenna azimuth offset, (**b**) antenna squint (**c**) and antenna tilt.

In order to derive the model, the biased antenna coordinated system ($x^{\prime },\;y^{\prime },\;z^{\prime }$) is calculated. The axes of the coordinated system (x,y,z) are rotated to get the new system ($x^{\prime },\;y^{\prime },\;z^{\prime }$) and the measured azimuth $\left(\theta_{m}\right)$ is calculated in the new system. Under the assumption that the deviation angles of the antenna axes are small (below 1°), the order of the rotation around each axis has a negligible influence in the model. The three rotation angles (Figure 1) correspond to the following bias parameters components:

- Azimuth offset $\left(\theta_{0}\right)$: it is generated with the rotation of the antenna in the horizontal plane. This offset is constant for all the targets of the sensor. This rotation is defined positive in counter-clockwise direction.
- Antenna squint $\left(s_{ant}\right)$: it is the bias produced due to the rotation in the orthogonal plane to the antenna boresight. This component produces an azimuth bias dependent on the target elevation (angle of line of sight respect to horizontal plane). This rotation is defined positive in counter-clockwise direction.
- Antenna tilt $\left(t_{ant}\right)$: produced due to the rotation of the plane of the 2D antenna pattern around the *x*-axis. As radars used in ATC applications do not measure the elevation, this bias has not consequences in the first order terms of the azimuth bias. This rotation is defined positive in clockwise direction.

The rotation matrix for each one of the axes are:

(4) $$R_{z}\left(\theta_{0}\right)=\left(\begin{array}{ccc} \cos\left(\theta_{0}\right) & \sin\left(\theta_{0}\right) & 0\\ -\sin\left(\theta_{0}\right) & \cos\left(\theta_{0}\right) & 0\\ 0 & 0 & 1 \end{array}\right)$$

(5) $$R_{x}\left(t_{ant}\right)=\left(\begin{array}{ccc} 1 & 0 & 0\\ 0 & \cos\left(t_{ant}\right) & -\sin\left(t_{ant}\right)\\ 0 & \sin\left(t_{ant}\right) & \cos\left(t_{ant}\right) \end{array}\right)$$

(6) $$R_{y}\left(s_{ant}\right)=\left(\begin{array}{ccc} \cos\left(s_{ant}\right) & 0 & -\sin\left(s_{ant}\right)\\ 0 & 1 & 0\\ \sin\left(s_{ant}\right) & 0 & \cos\left(s_{ant}\right) \end{array}\right)$$

Applying these rotations, the resultant coordinate system is:

(7) $$\left(\begin{array}{c} x^{\prime }\\ y^{\prime }\\ z^{\prime } \end{array}\right)=R_{z}\left(\theta_{0}\right)\cdot R_{x}\left(t_{ant}\right)\cdot R_{y}\left(s_{ant}\right)\cdot \left(\begin{array}{c} x\\ y\\ z \end{array}\right)$$

Due to the convention in geographical systems of the azimuth with the origin in the *y*-axis, positive in clockwise, the measured azimuth $\left(\theta_{m,ant}\right)$ is:

(8) $$\theta_{m,ant}=arctan\left(\frac{x^{\prime }}{y^{\prime }}\right)$$

These calculations are made under two assumptions: the error is only caused by the deviations of the antenna and this antenna can only acquire the targets that are in the 2D antenna main lobe. A multidimensional Taylor series on the bias variables $\theta_{0},s_{ant}\;\mathrm{and}\;t_{ant}$ is made to derive the following linearized approximation:

(9) $$\theta_{m,ant}\approx \theta +\theta_{0}-s_{ant}\tan\left(\varphi \right)\cos\left(\theta \right)+t_{ant}\tan\left(\varphi \right)\sin\left(\theta \right)\;+\left(\frac{1}{2}\sin\left(\theta \right)\cos\left(\theta \right)-\frac{\sin\left(\theta \right)\cos\left(\theta \right)}{\cos^{2}\left(\varphi \right)}\right)s_{ant}^{2}-\left(\frac{1}{2}\sin\left(\theta \right)\cos\left(\theta \right)-\frac{\sin\left(\theta \right)\cos\left(\theta \right)}{\cos^{2}\left(\varphi \right)}\right)t_{ant}^{2}\;+\left(\cos^{2}\left(\theta \right)-\frac{2\cos^{2}\left(\theta \right)}{\cos^{2}\left(\varphi \right)}+\frac{1}{\cos^{2}\left(\varphi \right)}\right)t_{ant}s_{ant}+\cdots$$

where φ is the ideal elevation of the aircraft. The variable θ may be particularized to zero in this case as the antenna only measures the aircraft that are into the antenna main lobe (θ≈0). Assuming that $\theta_{0},\;s_{ant}\;\mathrm{and}\;t_{ant}$ are small values then the higher order terms may be assumed to be negligible, resulting in:

(10) $$\theta_{m,ant}\approx \theta_{0}-s_{ant}\tan\left(\varphi \right)$$

The previous measure just contains the projection of the antenna deviation parameters in the azimuth component (θ≈0; noise and other bias terms are neglected for this calculation), resulting in the following additive azimuth bias term:

(11) $$\Delta \theta_{ant}=\theta_{0}-s_{ant}\tan\left(\varphi \right)$$

### 3.2. Axis Skew

**Figure 2:**
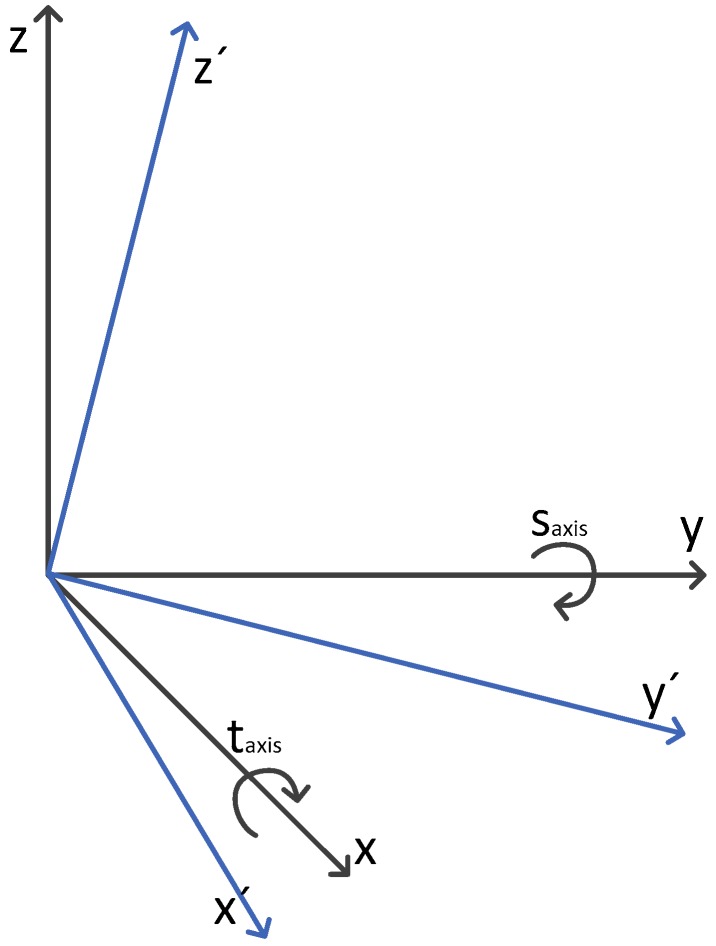
Skew of the rotation axis of the radar.

The axis skew is the inclination of the rotation axis. It causes an effect similar to the antenna deviation but in this case the bias also depends on the azimuth. For this model we use the local Cartesian coordinated system in the radar position: the *y*-axis pointing to the north and the *x*-axis pointing to the east both in the Earth horizontal plane. As in the previous subsection the biases of the azimuth can be calculated with rotations of the system axis as is represented in Figure 2.

In order to be coherent with the names of the rotations, the rotation in *y*-axis will be named axis squint and the rotation in *x*-axis will be named axis tilt. In this case, the tilt gives errors on the measures because it is the rotation in the *x*-axis and not in the orthogonal plane to the antenna boresight.

In this case the rotation matrix are:

(12) $$R_{x}\left(t_{axis}\right)=\left(\begin{array}{ccc} 1 & 0 & 0\\ 0 & \cos\left(t_{axis}\right) & -\sin\left(t_{axis}\right)\\ 0 & \sin\left(t_{axis}\right) & \cos\left(t_{axis}\right) \end{array}\right)$$

(13) $$R_{y}\left(s_{axis}\right)=\left(\begin{array}{ccc} \cos\left(s_{axis}\right) & 0 & -\sin\left(s_{axis}\right)\\ 0 & 1 & 0\\ \sin\left(s_{axis}\right) & 0 & \cos\left(s_{axis}\right) \end{array}\right)$$

The new coordinates system ($x^{\prime },\;y^{\prime },\;z^{\prime }$) is:

(14) $$\left(\begin{array}{c} x^{\prime }\\ y^{\prime }\\ z^{\prime } \end{array}\right)=R_{x}\left(t_{axis}\right)\cdot R_{y}\left(s_{axis}\right)\cdot \left(\begin{array}{c} x\\ y\\ z \end{array}\right)$$

The measured azimuth is calculated again $\left(\theta_{m,axis}=\arctan\left(\frac{x^{\prime }}{y^{\prime }}\right)\right)$ and the result is approximated linearizing with a multidimensional Taylor series on the bias variables $s_{ant}\;\mathrm{and}\;t_{ant}$. In this case the azimuth is not particularized to zero (θ≈0) because the reference axes are fixed to north and the ideal measurement can have any value of azimuth. The linear approximation, rejecting higher order terms in Taylor series is:

(15) $$\theta_{m,axis}\approx \theta +t_{axis}\tan\left(\varphi \right)\sin\left(\theta \right)-s_{axis}\tan\left(\varphi \right)\cos\left(\theta \right)$$

Therefore, the azimuth bias due to the axis skew is:

(16) $$\Delta \theta_{axis}=\theta_{m,axis}-\theta \approx t_{axis}\tan\left(\varphi \right)\sin\left(\theta \right)-s_{axis}\tan\left(\varphi \right)\cos\left(\theta \right)$$

**Figure 3:**
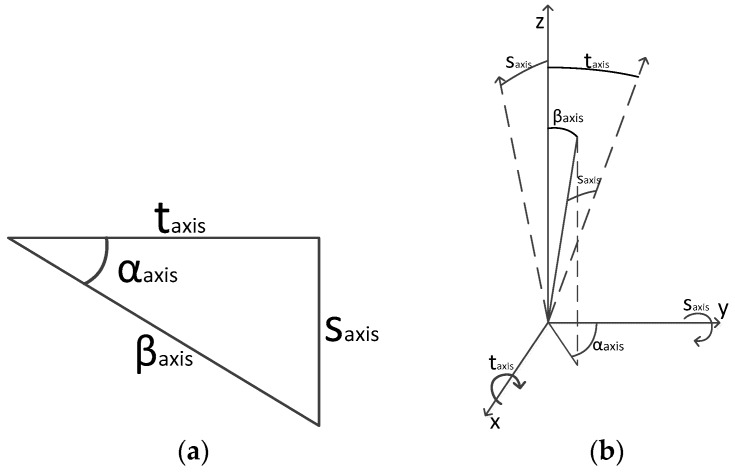
(**a**) Right triangle of the axis skew and (**b**) relation between the rotation of the axis and the skew of the rotation axis and its direction.

An alternative equivalent model to the one in (16) can be derived as follows. Under the assumption that the inclination of the axis is small, the spherical triangles with components $t_{axis}$ and $s_{axis}$ can be approximated by the right triangle of Figure 3, expressed in terms of $\beta_{axis}$ and $\alpha_{axis}$ where $\beta_{axis}$ represents the total skew and $\alpha_{axis}$ the direction where the axis is skewed. The new relation is:

(17) $$s_{axis}=\beta_{axis}\sin\left(\alpha_{axis}\right)\;t_{axis}=\beta_{axis}\cos\left(\alpha_{axis}\right)$$

Substituting (17) in (16) the final model could be written, after some minor algebra, as:

(18) $$\Delta \theta_{axis}=\beta_{axis}\sin\left(\theta -\alpha_{axis}\right)\tan\left(\varphi \right)$$

The azimuth offset due to this effect at the direction of the skew is zero, and it follows a sinusoidal law with azimuth. The model with $s_{axis}$ and $t_{axis}$ parameters in (16) is equivalent to the model with $\beta_{axis}$ and $\alpha_{axis}$ parameters in (18). The former model (16) results generally in better stability when used for the development for bias estimation algorithms.

### 3.3. Optical Encoder

In modern radars the azimuth of the antenna boresight is determined with an optical encoder that is allocated in the rotating radar shaft. In the installation of this encoder, small calibration errors can be made and these are transformed in azimuth biases. One of the errors is the misalignment between azimuth reference of the encoder and the axis, this is an azimuth offset. This error is the same kind that the azimuth offset of the antenna and the azimuth offset of the rotating axis, described in Section 3.1.

Other error to be considered is the eccentricity bias. Usually this is the encoder error with the biggest impact in the measures [19]. In the installation, the rotation axis (O) is not exactly placed in the geometric center (O’) of the encoder.

**Figure 4:**
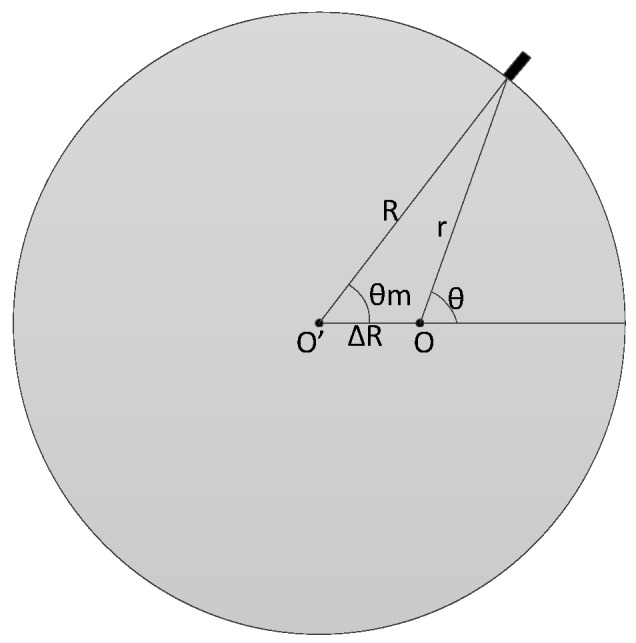
Optical encoder with eccentricity bias.

**Figure 5:**
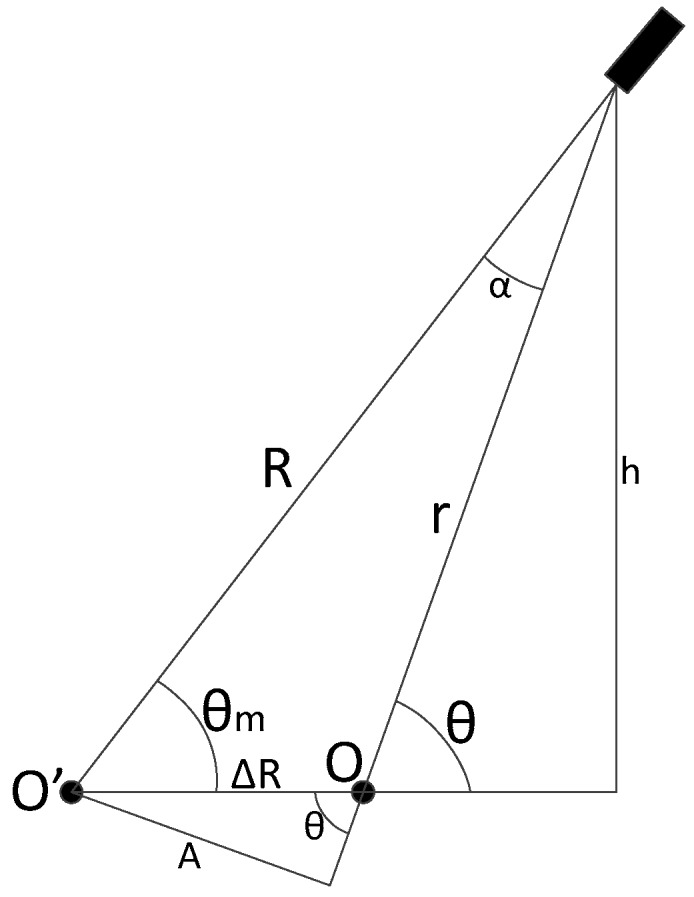
Resolution of the resultant triangle for eccentricity bias.

The resultant geometry is shown in the Figure 4, supposing that the difference of the centers is in the azimuth θ=0. Resolving the triangle shown in Figure 5 and making a deduction similar to [20,21] we have the following relation:

(19) $$\begin{array}{c} A=\Delta R\;\sin\left(\theta \right)\\ A=R\;\sin\left(\alpha \right) \end{array}\}=>\sin\left(\alpha \right)=\frac{\Delta R}{R}\sin\left(\theta \right)$$

where, ΔR is the eccentricity offset, *R* is radius of the encoder and *θ* and *α* are angles of the triangle shown in Figure 5.

Using the relation between sine and cosine for α ($\cos\left(\alpha \right)=\sqrt{1-\sin^{2}\left(\alpha \right)}$) we also have the following relation:

(20) $$\cos\left(\alpha \right)=\sqrt{1-{\left(\frac{\Delta R}{R}\right)}^{2}\sin^{2}\left(\theta \right)}=\frac{1}{R}\sqrt{1-\Delta R^{2}\sin^{2}\left(\theta \right)}$$

On the other hand, from Figure 5 we can also see:

(21) $$r=R\;\cos\left(\alpha \right)-\Delta R\cos\left(\theta \right)\;r=-\Delta R\cos\theta +\sqrt{R^{2}-\Delta R^{2}\sin^{2}\left(\theta \right)}$$

Finally:

(22) $$\begin{array}{c} h=R\;\sin\left(\theta_{m}\right)\\ h=r\;\sin\left(\theta \right) \end{array}\}=>\theta_{m}=arcsin\left(\frac{r}{R}\sin\left(\theta \right)\right)$$

The measured azimuth is also linearized with a Taylor series on the bias variable ΔR in order to get an approximation to model. Assuming that $\frac{\Delta R}{R}\ll 1$ the approximation of ${\left(\frac{\Delta R}{R}\right)}^{2}=0$ can be made. With these simplifications, the azimuth measurement can be approximated as:

(23) $$\theta_{m}\approx \theta -\frac{\Delta R}{R}\sin\left(\theta \right)$$

The second error produced by the optical encoder is due to the swash between the encoder and the horizontal plane. A small swash of the encoder produces that the projection of the circumference is an ellipse and the determination of the azimuth has errors when this azimuth does not point to the ellipse axis.

**Figure 6:**
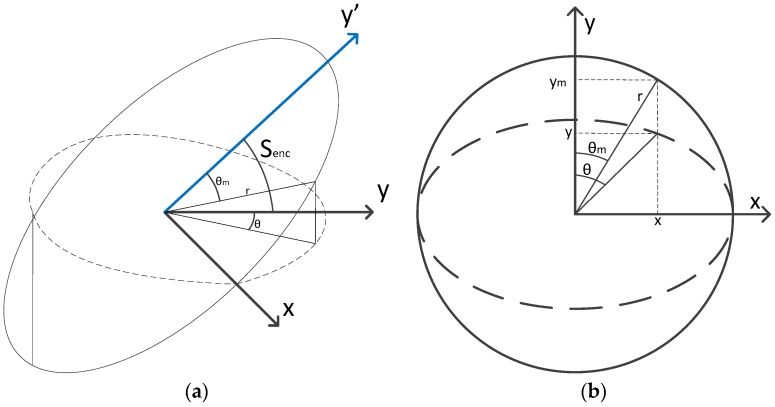
Swash of the optical encoder. (**a**) Schematic model of the optical encoder; (**b**) Mechanical encoder circumference and its projection on the horizontal plane.

In Figure 6a a schematic model of the optical encoder is represented. The circle line on the *x*-*y*’ plane represents the mechanical encoder and the dotted line on the *x*-*y* plane represents the projection of the rotated mechanical encoder circumference on the horizontal plane. The ideal azimuth is represented on the horizontal plane but the measured azimuth is obtained by the rotated encoder. In Figure 6b both mechanical encoder circumference and its projection on the horizontal plane are represented in the same plane in order to represent the 3D projection in a 2D figure to see the difference between the real azimuth and the measured azimuth. Following the methods used in [18] to convert geocentric latitude in reduced latitude we can obtain the relation between $\theta_{m}$ and θ. Using the next variables:

(24) $$x=r\;\sin\left(\theta_{m}\right)\;y_{m}=r\cos\left(\theta_{m}\right)\;y=y_{m}\cos\left(s_{enc}\right)$$

and solving the triangle for θ:

(25) $$\theta =atan\left(\frac{x}{y}\right)=atan\left(\frac{\tan\left(\theta_{m}\right)}{\cos\left(s_{enc}\right)}\right)$$

and then $\theta_{m}$ can be calculated as:

(26) $$\theta_{m}=atan\left(\cos\left(s_{enc}\right)\tan\left(\theta \right)\right)=atan\left(\cos\left(s_{enc}\right)\frac{x}{y}\right)$$

A Taylor series of order 2 on the bias variable $s_{enc}$ is made to get an approximation of the measure:

(27) $$\theta_{m}\approx \theta -\frac{1}{2}\cos\left(\theta \right)\sin\left(\theta \right)s_{enc}^{2}=\theta -\frac{s_{enc}^{2}}{4}\sin\left(2\theta \right)$$

In this Taylor series, the first order term is zero and the second order term is considered. As this term is raised to second power, with small values of $s_{enc}$ the value of the bias will be negligible. In both terms related with the encoder the error has been calculated supposing that the difference of the centers and the swash are in the azimuth θ=0. Supposing that in reality the azimuth of the difference of centers is $\alpha_{ecc}$ and the azimuth of the swash is $\alpha_{enc}$ the resultant encoder bias, comprising both the effects described in (23) and (27), is:

(28) $$\Delta \theta_{enc}=-\frac{s_{enc}^{2}}{4}\sin\left(2\theta -2\alpha_{enc}\right)-\frac{\Delta R}{R}\sin\left(\theta -\alpha_{ecc}\right)$$

An equivalent model can be derived by using trigonometric relations, in order to express all the biases with a linear structure similar to the one in Equation (16):

(29) $$\Delta \theta_{enc}=s_{enc,s}\sin\left(2\theta \right)+s_{enc,c}\cos\left(2\theta \right)+\Delta R_{y}\sin\left(\theta \right)-\Delta R_{x}\cos\left(\theta \right)$$

where:

(30) $$s_{enc,s}=\frac{s_{enc}^{2}\sin^{2}\left(\alpha_{enc}\right)-s_{enc}^{2}\cos^{2}\left(\alpha_{enc}\right)}{4}\;s_{enc,c}=s_{enc}^{2}\cos\left(\alpha_{enc}\right)\sin\left(\alpha_{enc}\right)$$

and $\Delta R_{y}$ and $\Delta R_{x}$ are the terms of the center deviation in the *y*-axis and in the *x*-axis.

### 3.4. Azimuth Bias Composition

The global azimuth bias model is a composition of the three bias elements previously discussed. Neglecting noise effects, in a radar, the measured azimuth of the antenna is the azimuth where the rotation axis is pointing plus the antenna bias. The azimuth of the rotation axis is the azimuth indicated by the encoder plus the axis bias. Finally, the azimuth indicated by the encoder is the ideal azimuth plus the encoder bias. With this:

(31) $$\theta_{enc}=\theta +\Delta \theta_{enc}\;\theta_{axis}=\theta_{enc}+\Delta \theta_{axis}\;\theta_{m}=\theta_{ant}=\theta_{axis}+\Delta \theta_{ant}$$

and then:

(32) $$\theta_{m}=\theta +\Delta \theta =\theta +\Delta \theta_{ant}+\Delta \theta_{axis}+\Delta \theta_{enc}$$

With this, the complete model for azimuth bias is:

(33) $$\Delta \theta =\Delta \theta_{ant}+\Delta \theta_{axis}+\Delta \theta_{enc}\;=\theta_{0}-s_{ant}\tan\left(\varphi \right)+\beta_{axis}\sin\left(\theta -\alpha_{axis}\right)\tan\left(\varphi \right)-\frac{1}{4}\sin\left(2\theta -2\alpha_{enc}\right)s_{enc}^{2}-\frac{\Delta R}{R}\sin\left(\theta -\alpha_{exc}\right)$$

This model should not be used in general in the bias estimation algorithms due to instability problems due to lack of linearity of some of part of the model, although its physical meaning is clear. The alternative equivalent model (preferred for bias estimation due to its linearity with respect to bias parameters) should be obtained composing (11), (16), (29) and (32), resulting:

(34) $$\Delta \theta =\theta_{0}-s_{ant}\tan\left(\varphi \right)+\left[t_{axis}\sin\left(\theta \right)-s_{axis}\cos\left(\theta \right)\right]\tan\left(\varphi \right)+s_{enc,s}\sin\left(2\theta \right)\;+s_{enc,c}\cos\left(2\theta \right)+\Delta R_{y}\sin\left(\theta \right)-\Delta R_{x}\cos\left(\theta \right)$$

## 4. Range Bias

Range biases are again due to several factors. The main factors that compose the biases are:

- Transponder delay: every aircraft has a different transponder delay (typically between −75 m and 75 m, usually assumed to be uniformly distributed). This aircraft dependent bias must be estimated for each aircraft in the tracking phase. The range model developed in this paper is for biases due to sensor and environment. As the estimation will be made with many targets, the mean of the delays of all the aircraft tends to be near zero. For more details on this bias term and methods to estimate it, see [8].
- Temporal reference error: radars measure the difference of time between the interrogation and the arrival of the response. Usually there is a small constant error in the reference time and it produces a constant error $\left(\rho_{0}\right)$ in the range estimation.
- Clock error: A small error in the clock calibration may produce a linear term in the range estimation [22]. In modern radar this term can be considered negligible due to the precision of the clocks.
- Propagation error: The conversion between time and distance is made using the speed of light in the ISA (International Standard Atmosphere) at mean sea level. However, the speed of light changes along the propagation path due to the change in the refraction index with altitude and weather conditions. Any change in the real speed of light will produce a range bias term ($\Delta \rho_{prop}$).

Considering the range biases terms previously explained, the range bias (Δρ, as defined (3)) results:

(35) $$\Delta \rho =\rho_{0}+\Delta \rho_{prop}$$

In cases when the clock has a bad calibration, the bias has additionally a linear term dependent on the clock error, and parameterized through a range gain factor ($\alpha_{clk}$), being the complete model:

(36) $$\Delta \rho =\rho_{0}+\alpha_{clk}\;\rho +\Delta \rho_{prop}$$

Next, we will focus on the propagation term.

### 4.1. Propagation Error

The index of refraction of the atmosphere changes with the altitude and this affects to the local speed of light. As the conversion between time and range is made with a constant speed of light, the determination of the position of the aircraft from the radar generally has biases (due to change of speed of light with altitude and weather conditions).

As the bias is produced by accumulated error in the speed of light along the propagation path, the range bias will be a function of the target range. In order to model the relation between the ideal range and the bias we will use a spherically stratified atmosphere. The exponential model of the refractivity [23] is used because it models the typical atmosphere variations in the rank of altitude used in ATC applications:

(37) $$N\left(h\right)=N_{s}exp\left(-\frac{h}{H}\right)$$

where *N*(*h*) is the refractivity at a given altitude (*h*) over mean sea level, *H* is a reference altitude (equal to 6950 m), and $N\left(0\right)=N_{s}$ (with typical values between 300 and 350). The relation between the refractivity and the index of refraction (*n*(*h*)) is:

(38) $$n\left(h\right)=1+N\left(h\right)\times 10^{-6}$$

The model in Equation (37) can be used also redefining altitude (h) to be referred to radar altitude, which just results in a change of the $N_{s}$ constant, to be now the refractivity at the radar altitude over mean sea level. This will be the altitude reference and refractivity model to be used for the rest of this section.

The height gradient of the index of refraction bends the ray, resulting in a slightly increased distance from that of the straight line used to model propagation on free space. Specifically, as it is deduced in [24] using a ray tracing procedure with the Snell’s law for spherically stratified media [25], the geometrical distance of the bent ray path may be calculated as:

(39) $$s\left(h_{t},\varphi_{0t}\right)=\int_{0}^{h_{t}}\frac{dh}{\sqrt{1-\left\{n_{0}\cos\left(\varphi_{0t}\right)\left[\frac{n\left(h\right)}{1+\frac{h}{R_{e}}}\right]\right\}}}$$

where $\varphi_{0t}$ is the elevation ray angle observed by the radar, $h_{t}$ is the target altitude relative to the radar, $n_{0}$ is the index of refraction at radar altitude, and $R_{e}$ is the Earth radius plus radar height. 

Additionally, the speed of light varies as it traverses different heights with different associated index of refraction. The effective distance (measured distance assuming constant speed of light, is affected by the propagation error), and can be calculated as proposed in [24]:

(40) $$r\left(h_{t},\varphi_{0t}\right)=\int_{0}^{h_{t}}\frac{n\left(h\right)dh}{\sqrt{1-\left\{n_{0}\cos\left(\varphi_{0t}\right)\left[\frac{n\left(h\right)}{1+\frac{h}{R_{e}}}\right]\right\}}}$$

In the following we will work with the approximation that the geometrical distance between the target and radar is almost equal to a straight-line (free-space) path length [24] ($\rho \approx s\left(h_{t},\varphi_{0t}\right)$). Then, the range bias due to propagation can be calculated as:

(41) $$\Delta \rho \left(h_{t},\varphi_{0t}\right)=r\left(h_{t},\varphi_{0t}\right)-s\left(h_{t},\varphi_{0t}\right)$$

**Figure 7:**
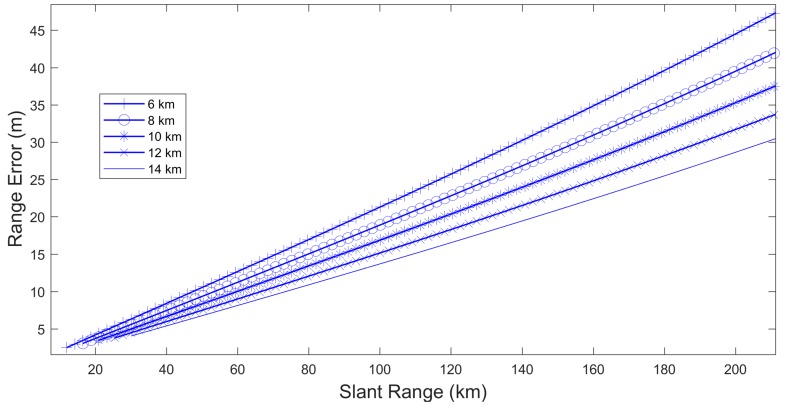
Range bias for aircraft at different altitudes.

Both distances (geometrical distance and effective distances), and their difference, can be numerically estimated for different values of $h_{t}$ and $\varphi_{0t}$. With these pairs we can get a table of range biases (Δρ) and geometrical distances (s). In these estimations we work with the approximation that the geometrical distance between the target and radar is equal to the ray path length [24]. Without this approximation the estimations are similar, but at distances lower than 20 Km the results of the biases are different. But in these places the total bias is very low (between 2 m and 6 m, depending on the altitude) and the approximation does not introduce significant error. The resultant range error is represented in the Figure 7, where each line represents the range error for a different altitude ($h_{t}$) as a function of slant range. 

As it is seen [26] two relevant characteristics can be observed. The first one is that the range error depends on the altitude of the aircraft. The second one is that a second-grade polynomial seems to be good enough to approximate the range error dependency with the slant range for every altitude [27]. Considering only this polynomial, the range error can be expressed as:

(42) $$\Delta \rho \left(\rho ,h\right)=\beta_{1}\left(h\right)\rho +\beta_{2}\left(h\right)\rho^{2}$$

where $\beta_{1}$(*h*) and $\beta_{2}\left(h\right)$ are polynomial coefficients, different for each altitude.

This model does not allow to make a good estimation of the parameters with opportunity traffic, because the number of aircraft in each altitude layer is reduced. We must find a model which allows the simultaneous use of aircraft measures at all altitudes in the estimation process.

A key idea in the derivation of such model is to search for decoupled range and altitude dependencies in Δρ(ρ,h):

(43) Δρ(ρ,h)=f(ρ)⋅g(h)

To do so, the quotient between a parabola in an altitude and the parabola at an arbitrary reference altitude ($h_{r}=$ 14,000 m in the following) for the same slant range is made:

(44) $$g\left(\rho ,h\right)=\frac{\Delta \rho \left(\rho ,h\right)}{\Delta \rho \left(\rho ,h_{r}\right)}$$

**Figure 8:**
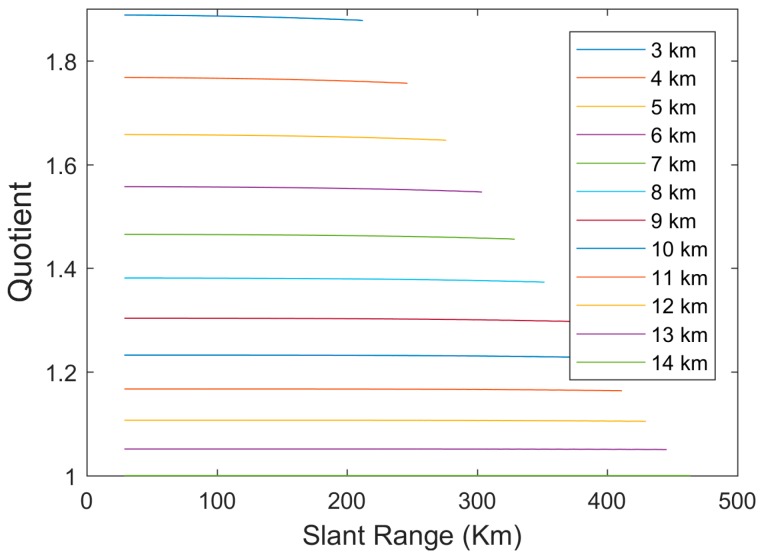
Quotient between range bias at an altitude and bias at 14,000 m for the same slant range.

This quotient is shown as a function of the slant range in Figure 8 and can be observed that for each altitude it is almost constant. The right end of the horizontal lines in this figure corresponds to the radio-wave propagation horizon. This result demonstrates that the range bias at a specific altitude is the range bias at the reference altitude multiplied by a constant, supporting Equation (43), as the dependency of g(ρ,h) with the slant-range is negligible (i.e., it could be substituted by g(h)), and f(ρ)⋅ would be directly $\Delta \rho \left(\rho ,h_{r}\right)$, a function not depending of h. From those ideas, we have that:

(45) $$f\left(\rho \right)=\beta_{1}\left(h_{r}\right)\rho +\beta_{2}\left(h_{r}\right)\rho^{2}=\alpha_{1}\rho +\alpha_{2}\rho^{2}$$

**Figure 9:**
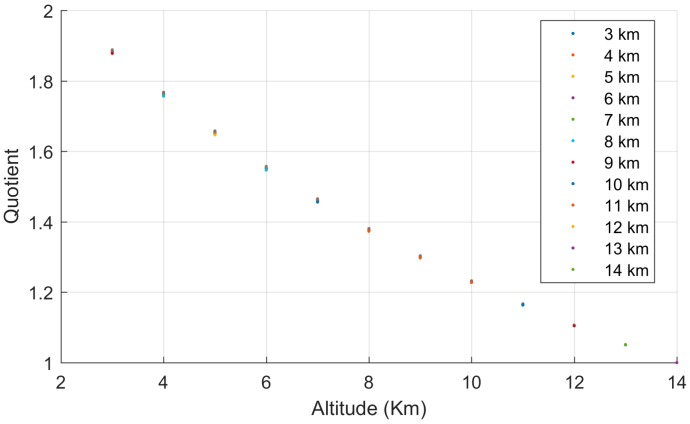
Ratio between range bias at an altitude and bias at reference altitude (14,000 m) for the same slant range as a function of altitude.

In order to derive g(h) we have represented in the Figure 9 the quotient between the range bias at an altitude and the bias at a reference altitude as a function of altitude. In this figure, it can be seen that a linear model is a good approximation. A second order polynomial would be an almost exact approximation but it implies the addition of another parameter that would make the convergence of the bias estimation processes slower with reduced accuracy gains.

Then *g*(*h*) can be modelled with a linear function normalized to reference altitude ($h_{r}$):

(46) $$g\left(h\right)=\frac{\Delta \rho \left(\rho ,h\right)}{\Delta \rho \left(\rho ,h_{r}\right)}\approx 1+\alpha_{3}\left(1-\frac{h}{h_{r}}\right)$$

Finally, the contribution of the tropospheric propagation to the range bias can be modelled, combining Equations (43), (45) and (46):

(47) $$\Delta \rho_{prop}=\left(\alpha_{1}\rho +\alpha_{2}\rho^{2}\right)\left[1+\alpha_{3}\left(1-\frac{h}{h_{r}}\right)\right]$$

### 4.2. Range Error Composition

The final range bias is the addition on the one hand of the propagation bias due to the atmosphere and on the other hand to the constant bias produced by the transponder delay and the error of reference time:

(48) $$\Delta \rho =\Delta \rho_{0}+\left(\alpha_{1}\rho +\alpha_{2}\rho^{2}\right)\left[1+\alpha_{3}\left(1-\frac{h_{t}}{h_{r}}\right)\right]$$

## 5. Altitude Bias

Civil surveillance radars used in ATC do not measure elevation of the targets. The reply of the aircraft transponder to the SSR interrogation codifies the altitude of the aircraft in the message (Mode C), completing the 3D position determination by the radar sensor. Aircraft altitudes may be of two different types:

- Geometric altitude: it is the Euclidean distance between the mean sea level (MSL) and the position of the aircraft.
- Barometric altitude: this altitude is calculated from the air pressure of the atmosphere at aircraft location. The conversion between pressure and altitude is made assuming International Standard Atmosphere (ISA) model (temperature 288.15 K and pressure 101,325 Pa, at MSL) [28]. Barometric altitude is the same that geometric altitude only when the atmospheric conditions are the ISA conditions.

Barometric altitude is used in ATC systems, because all aircraft must always measure the pressure to fly and its flight performance depends critically on this pressure, barometers are easy to be calibrated and vertical separation can be done easily using this magnitude. On board certified sensors for geometric altitude usable in all phases of flight (such as GPS) have only recently become widely available, and this information is not always available in the ground. All the surveillance systems send barometric altitude but only some of them can send the geometric altitude. Using the barometric altitude usually implies systematic errors in altitude determination that are almost equal for every aircraft and those depends on the pressure and temperature.

This altitude bias term, when the polar measure is transformed to the horizontal plane, introduces a systematic error in the 2D position. The projection of this bias will be negligible when the elevation of the aircraft is close to zero, but it will be an important factor when the aircraft is near to the sensor and the elevation is big. This situation is typical in some air traffic control systems, as that of a radar close to an airport with several others far away of that airport. Then the horizontal projection of the measures from the near radar are heavily distorted due to the systematic altitude errors.

**Figure 10:**
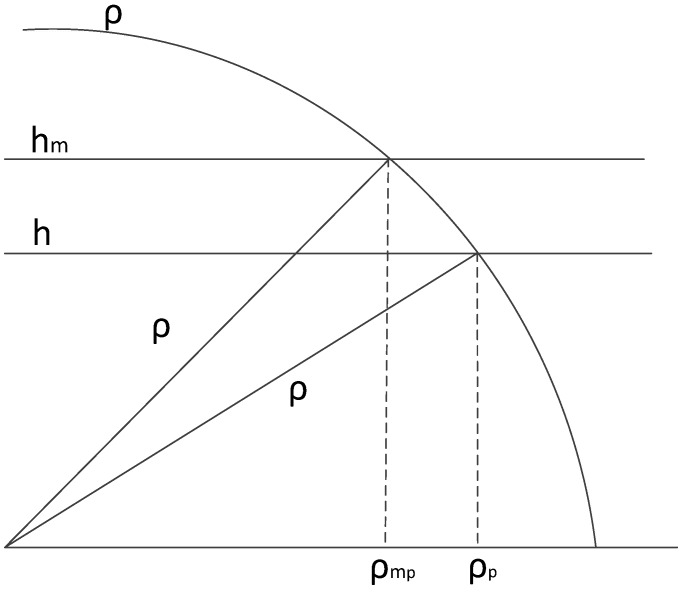
Difference between the projection in the slant range for the same range measure and different altitude.

The projection in the horizontal plane is implemented using the target elevation, as calculated in Equation (2). This equation is equivalent to search the cross point between the circle defined with radius ρ and the line defining the constant measured altitude. This is shown the Figure 10, where ρ is the measured range, *h* and $h_{m}$ are the ideal and measured altitude respectively. $\rho_{p}$ is the projected range calculated with the ideal altitude and $\rho_{mp}$ is the projected range calculated with the measured altitude.

In [28] a model of the atmosphere for its use in Air Traffic Management (ATM) trajectory prediction, based on ISA model, is defined. The ranges of altitudes of civil aircraft cover the troposphere and the lower stratosphere. The tropopause is the boundary between both layers. Hence there are two different models for different altitudes. In this model, the tropopause is allocated where the barometric altitude is 11,000 m. Below the tropopause the atmospheric model of barometric altitude is a non-linear function that depends on the temperature and the pressure at MSL. Above the tropopause, the model is a linear function because the gradient of temperature is zero.

In the model, the barometric altitude ($h_{p}$) is the measured altitude and the geometric altitude ($h_{g}$) will be the ideal altitude. The difference between both altitudes will be considered the altitude bias. The model proposed in [28] is:

Below tropopause:

(49) $$h_{g}=h_{p}-\Delta H_{P}+\frac{\Delta T}{\beta_{t}}\ln\left(\frac{T_{0}+\beta_{t}h_{p}}{T_{0}+\beta_{t}\Delta H_{p}}\right)$$

Above tropopause:

(50) $$h_{g}=h_{g,trop}+\frac{T_{0}+\Delta T+\beta_{t}h_{p,trop}}{T_{0}+\beta_{t}h_{p,trop}}\left(h_{p}-h_{p,trop}\right)$$

where $\Delta H_{P}$ is the barometric altitude offset with respect to ISA (which depends on the difference between current pressure and standard pressure at mean sea level), ΔT is the difference between current temperature and standard temperature at mean sea level, $T_{0}$ is the temperature of the standard atmosphere at mean sea level, $\beta_{t}$ is the temperature gradient and $h_{g,trop}$ is the geometric altitude where the barometric altitude is $h_{p,trop}=$ 11,000 m (which should be calculated with Equation (49) to guarantee geometric height continuity).

To reduce the impact of altitude biases in measurement projection in the horizontal plane ΔT and $\Delta H_{p}$ must be estimated and corrected. Next we will derive a parametric model for these terms. Note the altitude bias effect is important when the elevation of the aircraft is big (high altitude and short range). We will next fit and show the consistency of a linear model between the geometric altitude and the barometric altitude based on the previously presented model, resulting in:

Below tropopause: we may approximate linearly ln(1+x)≈x when x≈0. Using this approximation in $\ln\left(1+\frac{T_{0}+\beta_{t}h_{p}}{T_{0}+\beta_{t}\Delta H_{p}}-1\right)$ from (49) we can derive:

(51) $$h_{g}=h_{p}-\Delta H_{P}+\frac{\Delta T}{\beta_{t}}\left(\frac{T_{0}+\beta_{t}h_{p}-T_{0}+\beta_{t}\Delta H_{p}}{T_{0}+\beta_{t}\Delta H_{p}}\right)$$

After some algebra we have:

(52) $$h_{g}=\left(h_{p}-\Delta H_{P}\right)\left(1+\frac{\Delta T}{T_{0}+\beta_{t}\Delta H_{p}}\right)$$

and then we can solve for $h_{p}$, resulting:

(53) $$h_{p}=\frac{h_{g}}{1+\frac{\Delta T}{T_{0}+\beta_{t}\Delta H_{p}}}+\Delta H_{P}$$

Above tropopause: Regrouping the terms in Equation (50), and approximating $h_{g,trop}$ using (52), the following relation is obtained:

(54) $$h_{g}=h_{g,trop}+\left(1+\frac{\Delta T}{T_{0}+\beta_{t}h_{p,trop}}\right)\left(h_{p}-h_{p,trop}\right)$$

where:

(55) $$h_{g,trop}=\left(h_{p,trop}-\Delta H_{p}\right)\left(1+\frac{\Delta T}{T_{0}+\beta_{t}\Delta H_{p}}\right)$$

Then, we can solve again for $h_{p}$, resulting:

(56) $$h_{p}=\frac{h_{g}-h_{g,trop}}{1+\frac{\Delta T}{T_{0}+\beta_{t}h_{p,trop}}}+h_{p,trop}$$

In this section $h_{g}$ is always the ideal geometric altitude (called h in Equation (3)) and $h_{p}$ is the barometric altitude (measured by the barometer, called $h_{m}$ in Equation (3), assuming negligible altitude noise). Changing the notation to that of Equation (3) the resultant model is:

Below tropopause:

(57) $$h_{m}=\frac{h}{1+\frac{\Delta T}{T_{0}+\beta_{t}\Delta H_{p}}}+\Delta H_{p}$$

Above tropopause:

(58) $$h_{m}=\frac{h-h_{g,trop}}{1+\frac{\Delta T}{T_{0}+\beta_{t}h_{p,trop}}}+h_{p,trop}$$

**Figure 11:**
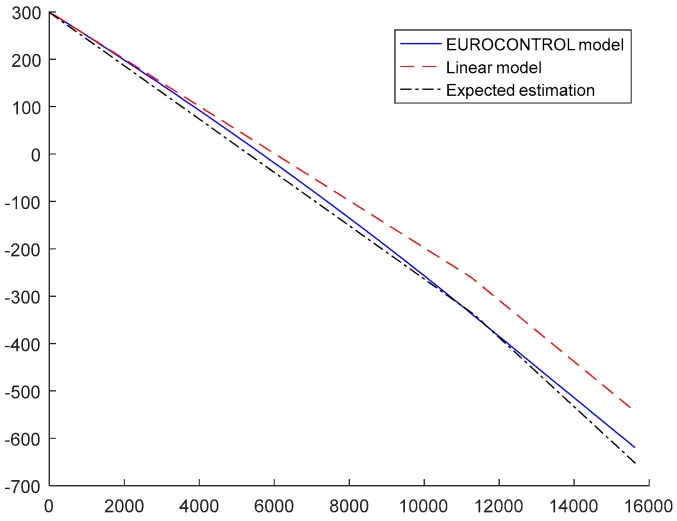
Representation of altitude bias for EUROCONTROL model, linearized model and expected estimation for $\Delta H_{p}$= 300 m and ΔT = 15°.

In both cases, the model has an offset height and a height gain. In Figure 11 the bias associated to the more exact EUROCONTROL model [28] in Equations (49) and (50), and the linearized model are represented (in Equations (57) and (58)) vs. the geometric altitude (h). Those two models use the same values for the parameters $\Delta H_{p}=300\;m$ and ΔT = 15°, and the linearization has very low error at low altitudes but at high altitudes the errors between both models can be around 100 m. Meanwhile, the black line (labelled expected estimation) represents a least squares regression of the EUROCONTROL model using the model in Equations (57) and (58) with parameters $\Delta H_{p}$ and ΔT. The optimal values of the estimated values, assuming uniform distribution of heights, are $\Delta H_{p}$= 298.72 m and ΔT = 17.06 °C. Although those are not the actual values used for the EUROCONTROL model, the simplified model is able to adjust quite finely to it, and therefore seems a promising model for altitude bias error estimation. If should be noted the purpose of this model is not obtaining a very accurate estimate of $\Delta H_{p}$ and ΔT, but to be able to approximate correctly the altitude bias relation with geometric height.

## 6. Bias Estimation with Opportunity Traffic

As the bias error varies slowly with time, the error model parameters must be estimated dynamically. In a radar network the main real time information source are the measures taken from the opportunity traffic and they are used for bias estimation. With the measures of a single radar the biases cannot be determined because there is not a reference in order to get the projection of the biases over the horizontal measurement plane. The projection of the biases depends on the position of the aircraft and the radar.

In this section, we will describe a method for the estimation of the bias parameters of a pair of radars with overlapped coverage. The purpose of the described method is not to describe an optimal/scalable process implementable in a real sensor network with more than two radars, but to analyze in the results section the adequacy of the proposed bias models and their effectiveness to remove systematic errors. More advanced methods may be found in the literature [6,7,8,9,13,14,15,16,17], and the application/extension of some of them will be part of our future research.

From the previous sections, bias model parameters of the *i*-th radar can be arranged in a 12D vector:

(59) $$b_{i}={\left[\Delta \theta_{0}\;s_{ant}\;t_{axis}\;s_{axis}\;\Delta R_{x}\;\Delta R_{y}\;s_{enc,s}\;s_{enc,c}\;\Delta \rho_{0}\;\alpha_{1}\;\alpha_{2}\;\alpha_{3}\right]}^{\prime }$$

On the other side, altitude bias terms due to the atmosphere conditions are common to every radar and they are included in other bias vector:

(60) $$b_{atm}={\left[\Delta H_{p}\;\Delta T\right]}^{\prime }$$

In our simple example method biases will be estimated with the difference of measurements (pseudomeasurements) of the same aircraft taken by different sensors at the same time. This pseudomeasurements will be obtained in the common plane subtracting the horizontal projection of the same aircraft measures. As in real scenarios the measurements of the radars are not synchronized the measurements of one of the sensors need to be interpolated to the time of the other radar, following a process as proposed in [7]. This interpolation is made between two measures from the same radar to the measure time from the other radar (between the times from the other two measures). Assuming that biases are small the projection of the bias terms may be approximated with a linearized function. With this linearization the measurement model for each of the plots projected in stereographic coordinates can be expressed as:

(61) $$X_{m,i}=\left(\begin{array}{c} x_{m,i}\\ y_{m,i} \end{array}\right)=\left(\begin{array}{c} x\\ y \end{array}\right)+H_{bi}b_{i}+H_{b,atm}b_{atm}$$

where (*x*,*y*) is the ideal horizontal position of the aircraft and $\left(x_{m,i},y_{m,i}\right)$ is the measured positon of the aircraft by the *i*-th radar while $H_{bi}$ and $H_{b,atm}$ are the bias projection matrices.

The projection matrices ($H_{bi}$ and $H_{b,atm}$) are a composition of the linearized change of coordinates from Equation (1), the linearization of the projection to the stereographic plane, and the linearization of the bias models for each of the bias components, so $H_{bi}$ and $H_{bi,atm}$ may be expressed as:

(62) $$H_{bi}=\left[\begin{array}{cc} \frac{dx}{d\Delta \theta_{0}} & \frac{dy}{d\Delta \theta_{0}}\\ \frac{dx}{ds_{ant}} & \frac{dy}{ds_{ant}}\\ \frac{dx}{dt_{axis}} & \frac{dy}{dt_{axis}}\\ \frac{dx}{ds_{axis}} & \frac{dy}{ds_{axis}}\\ \frac{dx}{d\Delta R_{x}} & \frac{dy}{d\Delta R_{x}}\\ \frac{dx}{d\Delta R_{y}} & \frac{dy}{d\Delta R_{y}}\\ \frac{dx}{ds_{enc,s}} & \frac{dy}{ds_{enc,s}}\\ \frac{dx}{ds_{enc,c}} & \frac{dy}{ds_{enc,c}}\\ \frac{dx}{d\Delta \rho_{0}} & \frac{dy}{d\Delta \rho_{0}}\\ \frac{dx}{d\alpha_{1}} & \frac{dy}{d\Delta \alpha_{1}}\\ \frac{dx}{d\alpha_{2}} & \frac{dy}{d\Delta \alpha_{2}}\\ \frac{dx}{d\alpha_{3}} & \frac{dy}{d\Delta \alpha_{3}} \end{array}\right]\;H_{bi,atm}=\left[\begin{array}{cc} \frac{dx}{d\Delta H_{P}} & \frac{dy}{d\Delta H_{P}}\\ \frac{dx}{d\Delta T} & \frac{dy}{d\Delta T} \end{array}\right]$$

### 6.1. Biases Values Initialization

The initial values of the biases will be estimated using a LSE method using a small number of measurement (for example the measurements received in an antenna scan period). As it is proposed in [6] a pseudomeasurement $\left(X_{b}\right)$ of the bias vector will be constructed and the (64) will be solved with the LSE method.

(63) $$X_{b}=X_{m,1}-{\hat{X}}_{m,2}$$

(64) $$X_{b}=H_{b}\;b+w$$

where $X_{m,1}$ is a measure from the radar 1, ${\hat{X}}_{m,2}$ is the interpolation from two measures from the radar 2 to the same time of $X_{m,1}$, *w* is the measurement white Gaussian noise projected to stereographic plane and:

(65) $$H_{b,i}=\left[H_{b1},\;-H_{b2},\;H_{b1,atm}-H_{b2,atm}\right]$$

(66) $$b=\left[\begin{array}{c} b_{1}\\ b_{2}\\ b_{atm} \end{array}\right]$$

where $H_{b,i}$ is the projection matrix of the *i*-th measurement. The biases are estimated using the pseudoinverse matrix [5]:

(67) $$b_{0}={\left[H^{\prime }R^{-1}H\right]}^{-1}H^{\prime }R^{-1}X_{b}$$

where:

(68) $$H=\left[\begin{array}{c} \begin{array}{c} H_{b,1}\\ \vdots \end{array}\\ H_{b,i}\\ \begin{array}{c} \vdots \\ H_{b,n} \end{array} \end{array}\right]R=diag\left(R_{1}\ldots R_{n}\right)$$

$R_{i}$ is the noise covariance matrix of each pseudomeasurement (it is the addition of the noise covariance matrix from $X_{m,1}$ and ${\hat{X}}_{m,2}$ of the *i*-th measurement) and n is the number of pseudomeasurements used in the initialization. The initial covariance matrix ($P_{0}$) used in the next section is calculated with the projection of the noise over the biases [5].

(69) $$P_{0}={\left(H^{\prime }R^{-1}H\right)}^{-1}$$

### 6.2. Recursive Bias Estimation

After initialization, the values of the biases are estimated recursively with an Extended Kalman Filter. Biases are considered constant, so the prediction matrix (Φ) is the identity matrix. Sub-index *k* indicates the temporal index of the state and the diacritic circumflex indicates that it is a predicted state. The predicted state and its covariance matrix at the time of the current pseudomeasurement are:

(70) $$\hat{b_{k}}=\Phi b_{k-1}\;\hat{P_{k}}=P_{k-1}\Phi P_{k-1}^{\prime }+Q_{k-1}$$

In simulated data where the biases are constant, the noise plant covariance matrix can be set to zero, but in real scenarios the biases will be quasi-constant and the *Q* matrix must be set with small values of covariance. After that the residual and its covariance matrix must be estimated:

(71) $$X_{b,k}=X_{m,1}-X_{m,2}\;r_{k}=X_{b,k}-H_{b,k}\hat{b_{k}}\;S_{k}=H_{b}\hat{P_{k}}H_{b}^{\prime }+R_{k}$$

With these previous values, the filter gain is calculated and son the updated state so the updated covariance of the filter are calculated:

(72) $$K_{k}=\hat{P_{k}}H_{b,k}^{\prime }S_{k}^{-1}\;b_{k}=\hat{b_{k}}+K_{k}r_{k}\;P_{k}=\left(I-K_{k}H_{b,k}\right)\;\hat{P_{k}}$$

### 6.3. Bias Observability Discussion

Ideally the observability of the bias parameters in the scenario can be tested with the matrix $\left[H^{\prime }R^{-1}H\right]$ where *H* and *R* is composed by all the $H_{b,k}$ and $R_{k}$ as in Equation (68). The bias parameters will be observable if this matrix is a positive definite matrix [15]. In the initialization phase this matrix is composed in order to calculate Equation (67). In the recursive bias estimation phase this matrix is never calculated but the observability can be tested composing this matrix with all the measurements. The dimensions of the resultant matrix becomes in an unaffordable computational load. The objective of this discussion is show the geometrical requirements (presented in the next paragraphs) that a radar scenario should have for the stability of the estimation algorithms. For a more rigorous mathematical analysis of the observability in radar registration the modified Fischer Information Matrix can be studied [29].

In the best case the targets have diversity in range, azimuth and altitude and the biases are perfectly determined. In many real cases the opportunity traffic is not uniformly distributed in the airspace as most of the aircraft are in the upper flight levels. LSE method tries to minimize the global errors for all the tracks. If most of the tracks are concentrated in the same zone, the method will estimate the bias model parameters to get the better approximation of the corrections for these tracks, but the estimated parameters could lead to a bad bias correction for the aircraft in areas with lower density traffic.

Another issue about the bias observability is the variability of the measurement values. If the sensors are widely separated in space, tracks with common coverage are concentrated in a small range of azimuth values. For example, in the azimuth bias model presented in Section 3, we may observe that the effect of the antenna squint and the axis squint are similar. The single difference between them is that axis squint depends on the azimuth of the target. If every target has similar azimuth, the effects of both bias terms are difficult to separate and their estimation is highly correlated. In this case, the position measures biases are well corrected for the tracks used in the estimation (inside common sensor coverage). But for other tracks with azimuth far from common coverage, there could be position errors because the azimuth bias dependence is not well estimated.

## 7. Results

In this section we will include results to show the performance improvement due to the use of our improved bias models both using simulated data (in Section 7.1) and real data (in Section 7.2). Also, the consistency of the estimator using this model will be assessed.

The evaluation of the results will be made evaluating the Root Mean Square (RMS) value of the difference between the positions of the measurements from different radars at the same time for the same target:

(73) $$error_{RMS}=\sqrt{\frac{1}{N}\sum_{i=1}^{N}\left({\left(x_{i,1}-x_{i,2}\right)}^{2}+{\left(y_{i,1}-y_{i,2}\right)}^{2}\right)}$$

where $\left(x_{i,1},y_{i,1}\right)$ and $\left(x_{i,2},y_{i,2}\right)$ are the *i*-th measures from first and second radar for the same target at the same time. This method is used because the evaluation of this model done principally with real data.

Another way to test the results for real data is to have access to ADS-B o WAM data that can be supposed unbiased compared with radar measurements. As the only available information is the measurements of the aircraft, only the errors of the corrected plots can be evaluated, but it is almost impossible know the real value of the biases. In route tracking in ATC it is more important the alignment between plot than the accuracy in position of the measurement.

For simulated data the value of the biases is known and the results are presented in convergence graphics (as a function of the number of samples used in the simulation).

### 7.1. Simulated Scenario Results

In order to test the observability of the different biases modelled in previous sections, the estimation method will be tested with a simulated scenario. In this scenario there are two radars in position (0, 0) and (50, 0) (in NM). The scenario has 1000 point uniformly distributed in a square area with a side of 400 NM centered at (0, 0). The maximum altitude of these points is 15,000 m.

In this case the simulated measurements are synchronized and the noise of the measurements are white Gaussian noise with zero mean with standard deviation of 75 m in range and 0.05° in azimuth. This test is made using Cartesian coordinates with the flat Earth model.

The measures obtained by the radars are biased using the previously developed models with all the parameters. The evaluation of the results is made using the RMS error in position of all the corrected measurement in Equation (73). As the ideal position of the points is known, the error will be calculated between the corrected measurement and the ideal position.

In this simulated scenario, the RMS error of the measured plots is 434.72 m and the RMS error of the corrected plots is 126.13 m. As in the simulated data the ideal position of the aircraft is known, we can measure the errors produced only by the noise. In this case, the RMS error produced just by the noise is 120.08 m. As the RMS error of the corrected plots is almost the RMS error produced by the noise of the measurements, it is proved the observability of the model when the plots are well distributed. With a bigger number of samples, the RMS error of the estimation is nearer to the RMS error produced by the noise.

In the previous simulated scenario the bias parameter used in the generation of the simulated position are shown in Table 1. The next paragraphs will show the estimation of some parameters using different models but always generating the simulated plots with the complete model.

In the evaluation of the simulated data, the complete model with the values indicated in Table 1 has been used in the generation of the plots, but different bias models with more or less parameters have been used in the estimation. The next figures shows the mean estimated value of the biases with a blue line and the mean plus/minus the standard deviation of the estimation with a dotted red line.

**Figure 12:**
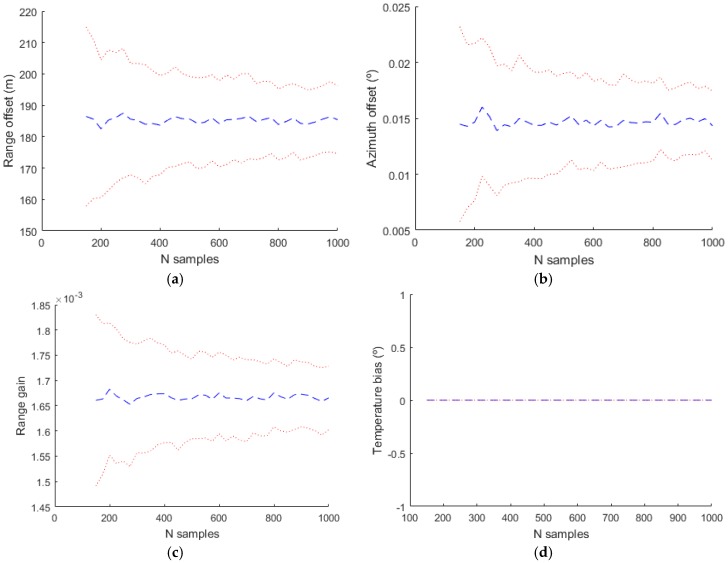
Estimated bias values for Radar 1 using only the basic model ($\Delta \rho_{0}$, $\alpha_{1}$ and $\theta_{0}$). (**a**) Range offset; (**b**) Azimuth offset; (**c**) Range gain; (**d**) Temperature bias.

**Figure 13:**
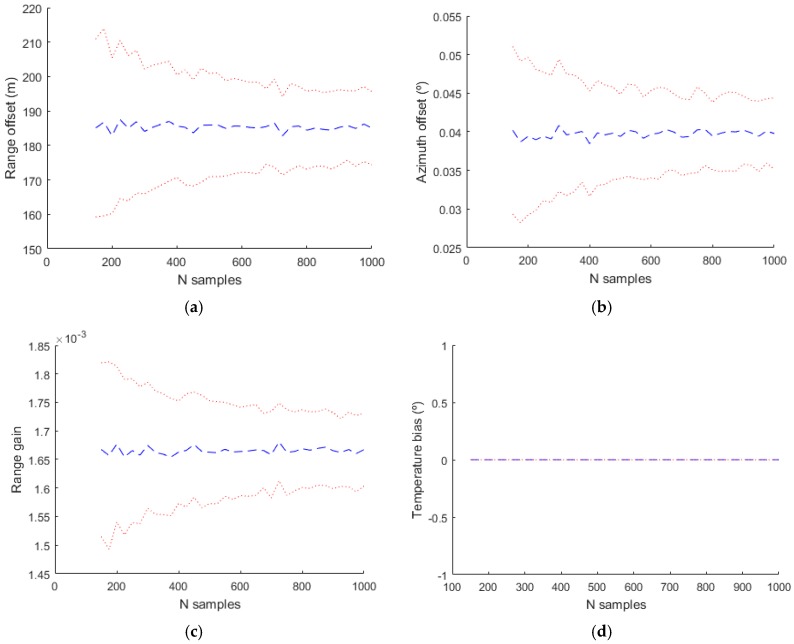
Estimated bias values for Radar 1 using only the model with the parameters $\Delta \rho_{0}$, $\alpha_{1}$, $\theta_{0}$,$s_{ant}$, $\beta_{axis}$ and $\alpha_{axis}$. (**a**) Range offset; (**b**) Azimuth offset; (**c**) Range gain; (**d**) Temperature bias.

The basic model used classically in the bias estimation literature uses only $\Delta \rho_{0}$, $\alpha_{1}$ and $\theta_{0}$. The obtained results are shown in Figure 12. The temperature bias isn´t estimated with this model and in the figure corresponding to this bias the value and the deviation is zero. With a more complex model where the parameters used in the estimation are $\Delta \rho_{0}$, $\alpha_{1}$, $\theta_{0}$, $s_{ant}$, $\beta_{axis}$ and $\alpha_{axis}$ the estimated values are shown in Figure 13.

**Figure 14:**
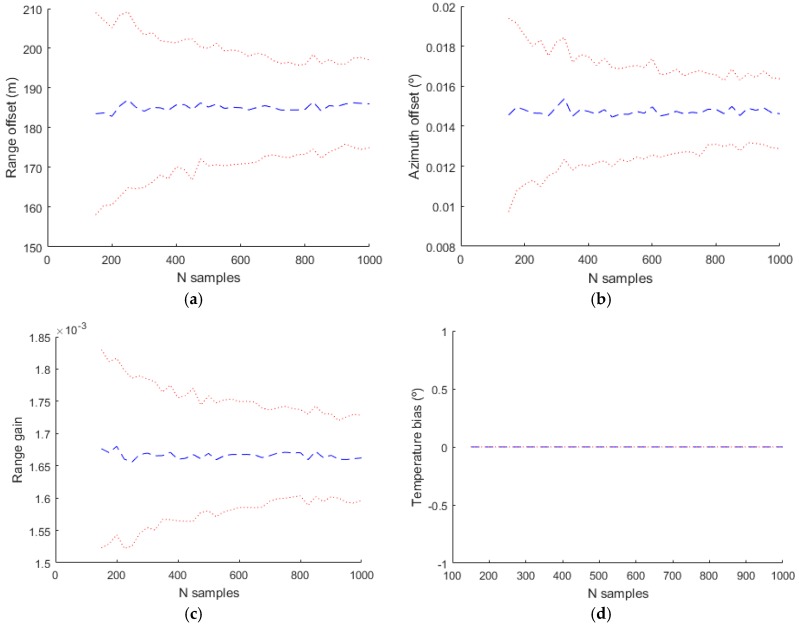
Estimated bias values for Radar 1 using only the model with the parameters $\Delta \rho_{0}$, $\alpha_{1}$, $\theta_{0}$, $\beta_{enc},$ $\alpha_{enc}$ ΔR/R and $\alpha_{exc}$. (**a**) Range offset; (**b**) Azimuth offset; (**c**) Range gain; (**d**) Temperature bias.

Estimating different parameters than in the previous simulation ($\Delta \rho_{0}$, $\alpha_{1}$, $\theta_{0}$, $\beta_{enc},$ $\alpha_{enc}$ ΔR/R and $\alpha_{exc}$) the results are slightly different as it can be seen in Figure 14. In these three simulations it can be seen that the range and azimuth estimations are different from the real values. This is due to the fact that there are parameters that affect range and azimuth that aren´t included in the estimation model and the included parameters values compensate the effect of the not included parameters.

**Figure 15:**
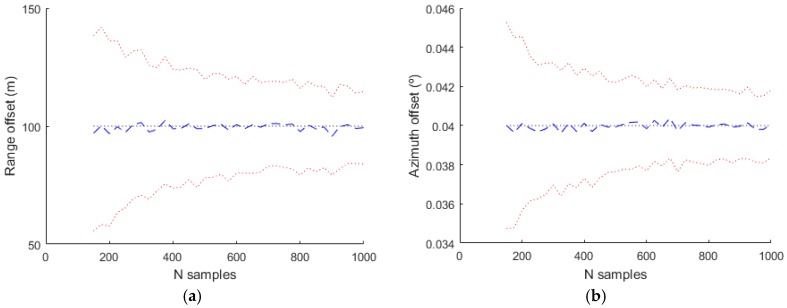

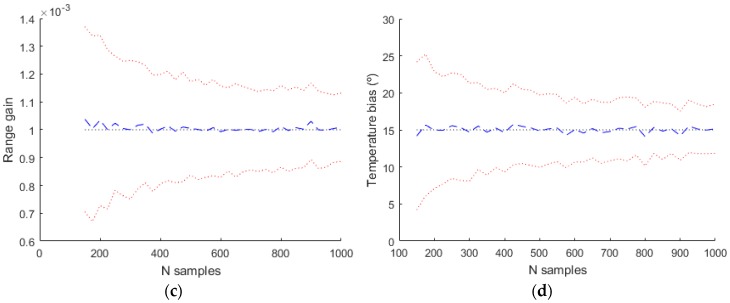
Estimated bias values for Radar 1 using the complete model. (**a**) Range offset; (**b**) Azimuth offset; (**c**) Range gain; (**d**) Temperature bias.

Finally Figure 15 shows the estimated values for the complete model used in the generation. In this case all the parameters are estimated and the result are near to the real values (shown with a black dotted line).

### 7.2. Real Data Results

The models have been tested with data from two real radars. The maximum range of both radars is bigger than 150 NM and the separation between both radars is 52 NM. This configuration gives enough common coverage and avoids observability problems due to the sensor separation. Radar measurements have been transformed to local Cartesian coordinates and projected to a common stereographic plane. The center of the stereographic projection (point (0, 0) in Figure 12) is far away from the radars in order to evaluate if the models work with the rotation, translation and scaling produced by the stereographic projection.

**Figure 16:**
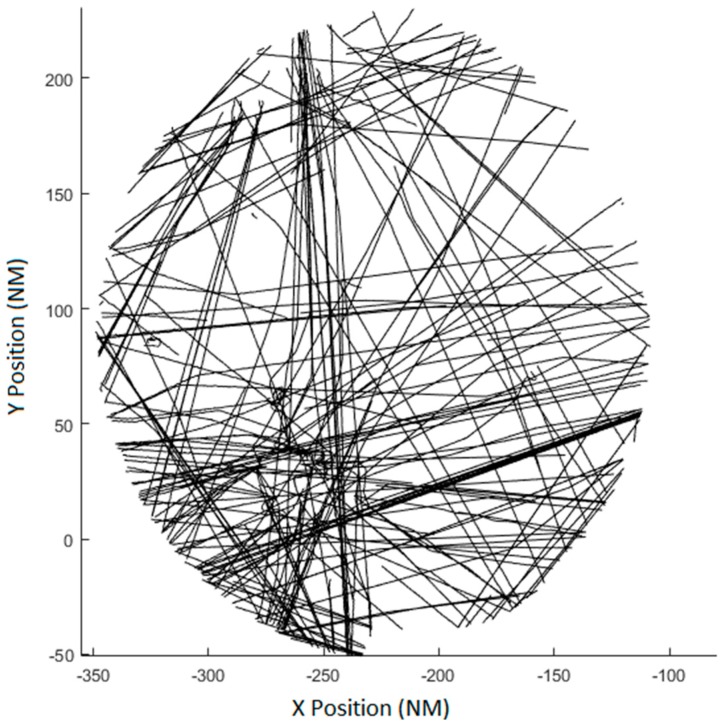
Real scenario with 227 tracks measured by two radars.

The real scenario has 227 aircraft distributed in the airspace in different flight phases. As it can be seen in Figure 16 tracks are not uniformly distributed in the airspace. The major part of the measures is concentrated in the bottom-left corner of the common radar coverage. As the algorithm minimizes the mean square error the measurement of this corner will be better aligned than the measurements from other parts of the scenario, as described in Section 6.3.

As the biases values are not known, the performance of the model will be evaluated estimating the RMS value of the deviation in measured position of the same track at the same time from two different radars (73). The evaluation will compare the deviation of the positions for all the measures in the common coverage. The RMS error of the measurement noise of the radar is 160.95 m for the first radar and 88.92 m for the second radar. The RMS value of the difference of uncorrected measured positions is 532.45 m.

### 7.3. Basic Model

**Figure 17:**
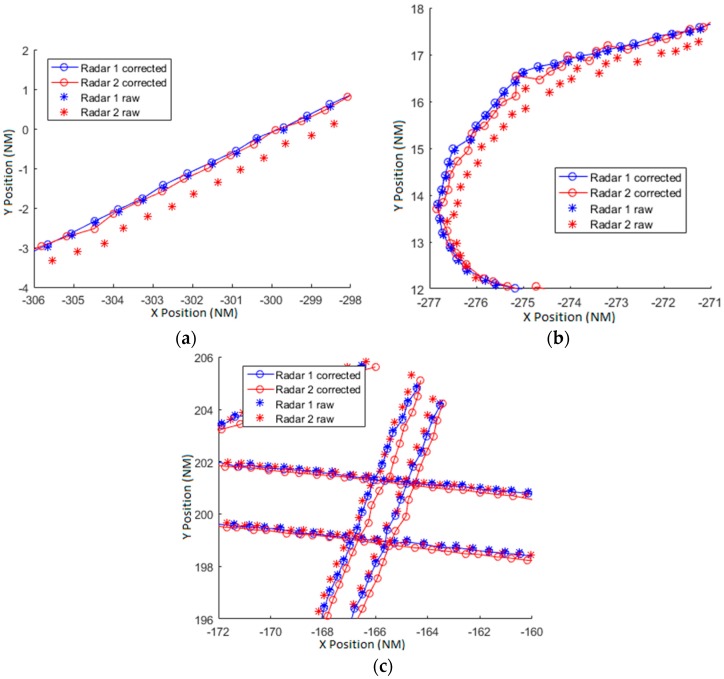
(**a**) Straight trajectory; (**b**) Take off trajectory corrected with basic model; (**c**) Several trajectories allocated at the upper-right corner of the scenario corrected with the basic model.

In [6,7] a simplified bias model is used for range and azimuth without bias in altitude. The biases used in this estimation are the range offset ($\Delta \rho_{0})$, the range gain ($\alpha_{1}$) and the azimuth offset ($\Delta \theta_{0}$). With these three parameters the majority of the error produced by the biases is corrected. With these models, the deviation of the bias errors $\sigma_{b}$ is reduced up to 286.60 m obtained with Equation (73). In Figure 17a,b two illustrative trajectories are represented. The asterisks mark the measured position and the circles mark the corrected position. Both trajectories are allocated in the bottom left corner of the scenario and the raw plots of radar 2 are displaced in azimuth in counter-clockwise direction. The algorithm corrects the plots and displaces the plots towards the correct azimuth. In Figure 17c several trajectories placed in top-right corner (low density traffic area) are represented. Raw plots are aligned and the corrected plots are displaced clockwise. In this scenario is easy to see that the simple model is not good enough to estimate and align the plots of the radars for the whole radar coverage. In this scenario, the trajectories of the Figure 17a,b are separated almost 180° in azimuth from trajectories of the Figure 17c. The azimuth dependence of the biases is clearly shown in these figures. This dependence is included in the complete azimuth bias model of Equation (34).

### 7.4. Complete Model

Next, we will show the results with variations of the complete model developed in this paper. Several configurations of the model have been used with the objective to test the final correction contribution of each parameter of the model. In Table 2 the error deviation due to the biases is expressed for different configurations of the models (in the first column the bias parameters used in each configuration are indicated). The first row is the basic model from the previous section. Second row estimates the biases due to the deviation of the antenna and the rotation axis. Third row estimates the biases produced by the encoder. Finally the fourth row estimates all the biases presented in the model.

**Figure 18:**
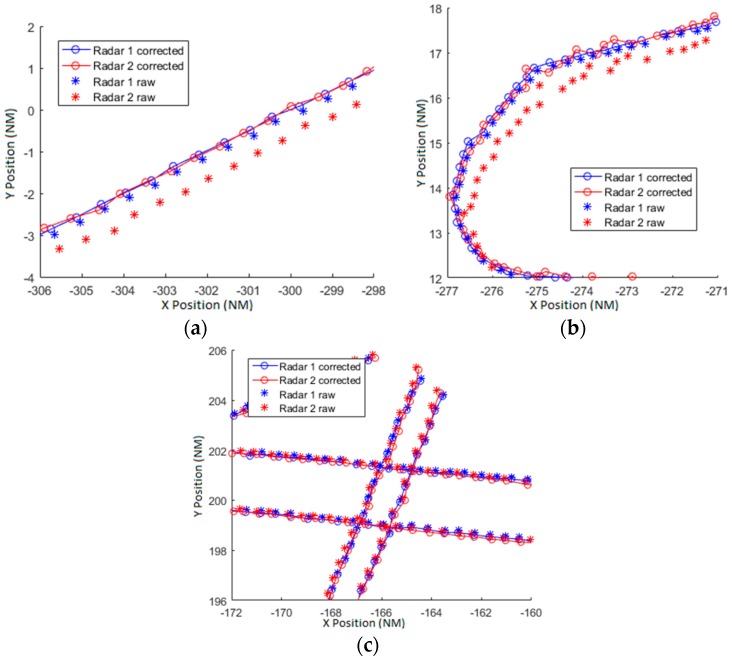
(**a**) Straight trajectory (**b**) Take off trajectory corrected with basic model (**c**) Several trajectories allocated at the upper-right corner of the scenario corrected with the complete model.

The encoder eccentricity seems to be a very important parameter in the bias estimation. For example if the encoder has a diameter of 25 cm and an error in the rotation center of 0.25 mm produces a maximum bias of 200 m at a range of 200 km. As could be expected, the estimation with all the parameters is the best one and in the Figure 18 (equivalent to Figure 17) the corrected tracks are shown. In this case, as the complete model has azimuth dependent biases, every track in every azimuth is better corrected in comparison with the simple model. But even reduced parameters bias models result in relevant accuracy gains with respect to the usual basic model.

The algorithm determines bias parameters for both radars to minimize the mean squared error between the ideal position and the corrected position. As consequence of results we should conclude that (locally in the in the shown areas) the radar 1 is better aligned to ideal position than radar 2, although measures from both radars are corrected.

## 8. Conclusions

A complete model for radar biases in ATC has been developed in the paper considering both mechanical errors in the radar installation and errors in the physical propagation and atmospheric parameters. Typically only a basic bias model is considered in bias estimation and correction. As has been demonstrated with our results, with these simplified model parameters the major part of the errors can be corrected. In real scenarios the errors of the measures cannot be approximated only with the basic bias model. In online applications where the execution time is an important feature, the simple model reduces the major part of the errors. In offline applications (such as [30]) where the accuracy of the estimations is more relevant than the execution time, the number of estimated parameters can be increased reducing the final bias error. As the number of parameters is increased, the degrees of freedom to interpolate generic bias patterns is increased too.

The bias estimation methods in general minimize the MSE and are better adjusted where the traffic density is bigger. With the basic model the biases are only adjusted to these higher density areas, leaving lower traffic density with badly corrected measures. With the complete biases model there are more degrees of freedom and the estimations will be better adjusted for areas where there is lower density.

Analyzing the results using different bias configurations (estimation with different bias parameters) it can be observed that the basic biases will correct the major part of the systematic errors. The encoder eccentricity has a significant effect on the azimuth that does not depend on the target elevation and the use of this parameter will align the plots better than with the simple model. Also, trajectories near to one of the radars are highly affected by altitude errors.

For future work two topics derived from this paper can be studied. On the one hand the distribution of the plots seems to be an important topic. An estimation method will be studied in order to try to make the density of the plots in a given area not so critical for the precision of the estimations. On the other hand, the parametrized model developed in this paper assumes that the atmospheric conditions are constant in all the airspace but the temperature and pressure at MSL change slowly with position. In the future we will also study the extension of the model with slow changing curves for pressure and temperature, and the potential associated improvements. Also, the estimation of transponder error, maybe exploiting ideas similar to the ones in [8] will be addressed.

## Figures and Tables

**Table 1:** Values of the biases used in the simulation for the radar 1.

Parameters	RMS Error
$\Delta \rho_{0}$	100 m
$\alpha_{1}$	10^−3^ m/m
$\alpha_{2}$	10^−9^ m/m^2^
$\alpha_{3}$	1.15
$\theta_{0}$	0.04°
$s_{ant}$	0.5°
$\beta_{axis}$	0.4°
$\alpha_{axis}$	45°
$\beta_{enc}$	0.5°
$\alpha_{enc}$	90°
ΔR/R	5 × 10^−4^
$\alpha_{exc}$	45°
$\Delta H_{p}$	−500 m
ΔT	15 °C

**Table 2:** Bias error deviation for different configuration of the model parameters.

Parameters	RMS Error
Without correction	532.45 m
$\left[\Delta \rho ,\alpha_{1},\Delta \theta \right]$ (basic model)	286.60 m
$\left[\Delta \rho ,\alpha_{1},\Delta \theta ,s_{ant},\beta_{axis},\alpha_{axis}\right]$	260.65 m
$\left[\Delta \rho ,\alpha_{1},\Delta \theta ,s_{enc},\alpha_{enc},\Delta R/R,\;\alpha_{ecc}\right]$	248.22 m
$\left[\Delta \rho ,\alpha_{1},\alpha_{2},\alpha_{3},s_{ant},s_{axis},t_{axis},\Delta \theta ,s_{enc},\alpha_{enc},\Delta R/R,\;\alpha_{ecc},\Delta H_{P},\Delta T\right]$	242.19 m
